# Changes and Rate of Change in Neutrophil-Lymphocyte Ratio (∆NLR) as an Early Prognostic Marker for the Severity of Outcomes in Patients With COVID-19 and Its Applicability in Other Viral and Bacterial Diseases

**DOI:** 10.7759/cureus.41774

**Published:** 2023-07-12

**Authors:** Nandana Jasti, Lakshmikanth Reddy MN, Naveen Kumar Pothireddy, Meghna R Sankepalli, Ganshyam M Jagathkar, Uday Pratap Singh

**Affiliations:** 1 Internal Medicine, Medicover Hospital, Hyderabad, IND; 2 Pulmonology, Medicover Hospital, Hyderabad, IND; 3 Intensive Care Unit, Medicover Hospital, Hyderabad, IND; 4 Oral Medicine, Medeva, Delhi, IND

**Keywords:** prediction model, overall severity, d-dimer, delta neutrophil lymphocyte ratio, covid-19

## Abstract

Introduction: COVID-19 is a global pandemic that has spread rapidly and resulted in numerous deaths worldwide. Many inflammatory markers such as neutrophil-lymphocyte ratio (NLR), D-dimer, serum ferritin, C-reactive protein (CRP), and interleukin-6 (IL-6) were used for the diagnosis and prognosis of COVID-19.

Methods: We have proposed using Delta NLR (0-48 hours) (∆NLR) as an early diagnostic marker for COVID-19 and other inflammatory disorders. We have created a prediction model based on six variables: overall severity, death, shifting to the ICU, length of stay, oxygen requirement, and ventilator support. Prediction models help us prepare for future pandemics through early diagnosis and management.

Results: A total of 1,865 patient records were retrieved from the database. The final sample available for analysis was 461. Change in NLR or ∆NLR was significant for all the models (except for length of stay) created by logistic regression.

Conclusion: An independent predictor of the poor prognosis of COVID-19 is the severity of the disease in the initial one or two days. ∆NLR is a unique marker, and its scope of use in other disorders’ prognoses must be further researched. The prediction models also help us in decision-making strategies and also prepare us for future pandemics.

## Introduction

The novel coronavirus (COVID-19) has been a rapidly spreading disease since the end of 2019, resulting in a global pandemic [1]. The illness spectrum varies, with the majority of instances being asymptomatic, mild, and self-limiting respiratory tract infection to severe pneumonia with acute respiratory distress syndrome and multiorgan dysfunction [2]. The first instances of COVID-19 appeared in China in 2019, and the WHO then proclaimed it a worldwide pandemic in March 2020 [2]. Globally, as of March 2023, there have been 758,390,564 confirmed cases of COVID-19, including 6,859,093 deaths, reported to WHO [3]. About 44.7 million cases are detected in India, with 5.31 lakh deaths, according to the Johns Hopkins University Center for Systems Science and Engineering COVID-19 data [4].

A severe form of COVID-19 develops mostly due to hyperinflammatory reactions [5]. Rapid viral multiplication, cellular damage, and inflammatory responses attract macrophages and monocytes that produce cytokines and chemokines [6] which cause cytokine storms and hyperactive immune responses [7]. This inflammatory response can be used to assess the degree and activity of the disease. Serum ferritin, C-reactive protein (CRP), D-dimer, and interleukin-6 (IL-6) are examples of inflammatory markers that have been shown to be strongly linked to the high risks of developing severe COVID-19. One of the indicators of inflammatory response is the neutrophil-lymphocyte ratio (NLR) [8,9], which is the ratio between the number of neutrophils and a number of lymphocytes, usually from a peripheral blood sample.

These inflammatory markers have also been used in assessing the severity of many other disorders. They are used as a predictor of prognosis in patients with cardiovascular disease [10,11], cancers [12,13], sepsis [14,15], bacteremia [16], viral infection [16], and psychiatry disorders [17,18].

NLR is used as a marker of inflammation first reported by a study in 2001 in which marked elevation of neutrophil counts and deep decline in lymphocyte counts were observed in patients with septic shock, hemorrhagic shock, multiple trauma, pancreatitis [19], autoimmune diseases [20], and tuberculosis [21]. NLR is also used as a marker in cardiovascular disease, various cancers, psychiatric disorders, and sepsis and to gauge the severity of infectious illnesses [22]. This measure has superior sensitivity and specificity than the WBC count [23].

In acute and chronic illnesses, NLR can predict disease severity in the initial stages of the disease regardless of the etiology, such as infection, autoimmune, or malignancy. There have been numerous studies that have documented the performance of NLR in comparison to other inflammatory markers, such as creatine phosphokinase, CRP, etc. [24,25]. However, there is still no concrete evidence that could highlight whether NLR is as good a predictor as others or not, and more importantly, a recently published paper identifies the role of NLR to be of limited applicability in predicting disease severity [26].

Delta NLR (∆NLR), defined as the difference between baseline and follow-up NLR, is considered a better predictor and is used in predicting the severity of various disorders. However, only a few research studies have documented the difference in NLR from baseline to day 1 or day 2 in accurately predicting disease severity [27]. ∆NLR (i.e., the change in NLR values over a period of time) has been used for assessing and predicting disease severity in COVID as well as many other disorders (cancers [28], cardiovascular disorders [29], renal disorders [30], etc.).

This study aims to determine if 24-hour and 48-hour ∆NLR can be used to estimate the outcomes, disease severity, and resources needed for managing patients with COVID-19.

## Materials and methods

Study participants and data collection

Records of patients admitted to the hospital wards and ICU of Medicover Hospital, Hyderabad (internal medicine, pulmonology, and critical care), for management of COVID-19 were retrieved from the hospital database starting from January 2021 to June 2021. This time span overlaps with the second wave of COVID-19 in India. Data related to patient demographics, medical history, complaints at presentation, laboratory values, and outcomes were captured.

The following parameters were mandatory requirements: age, gender, vitals (height, weight, oxygen saturation (SpO2), pulse rate, respiratory rate, systolic blood pressure (SBP), diastolic blood pressure (DBP), random blood sugar, medical comorbidities, COVID-19 (severity, symptoms, and management), laboratory investigations, neutrophils count, lymphocytes count, NLR every 24 hours, glycosylated hemoglobin (HbA1c), D-dimer, and CRP at baseline and when needed by the treating physician.

Other considered parameters were complete recovery, death, COVID-19-related organ dysfunction, admission to the ICU, shift to ICU during the stay, ventilator support required, and oxygen requirement.

The data available of all the patients admitted to the hospital during the second wave of COVID-19 were included in the study. The data was anonymized at the source before collating.

All patients older than 18 years and admitted for the treatment of COVID-19 during the second wave were eligible to participate in the study. Patients who were lesser than 18 years of age, who were COVID-19 positive but admitted for management of another disorder, or COVID-19 positive patients with any concomitant autoimmune disorders were also excluded.

Categorization of the severity of COVID-19

The severity of COVID-19 was assessed using six variables: death as the final outcome, shifting to ICU during the stay, oxygen requirement, the requirement for ventilator support, length of stay, and direct admission to ICU. The categorization is explained in detail in Table 1. Binary severity variables were created by using all the available variables.

**Table 1:** Variables for assessing COVID-19 severity ICU: intensive care unit, NSC-19: not severe COVID-19, SC-19: severe COVID-19, NIV: noninvasive ventilation, CPAP: continuous positive airway pressure, BIPAP: bilevel positive airway pressure, HCM: high-concentration mask, HFNC: high-flow nasal cannula

Calculation of overall severity score			Weightage (%)
Severity variable	NSC-19	SC-19	
Death	Survival of patient	Expiry of patient	30
Shifting to ICU during the stay	No shifting to ICU from the ward needed	Patient shifted to ICU from initial admission to ward	15
Oxygen requirement	0: no oxygen. 1: <4/nasal cannula. 2A: 4 to 15/face mask. 2B: 4 to 15/face mask with non-rebreathers (HCM)	3A: NIV/CPAP or BIPAP. 3B: NIV/HFNC. 4: ventilator	15
Requirement for ventilator support	Ventilator support not needed throughout the stay	Ventilator support needed at least once in four days and/or at the discharge time	15
Direct admission to ICU	Patient not admitted to ICU at admission	Patient admitted to ICU directly on admission	15
Length of stay	Less than or equal to the 60th percentile of the length of stay of all patients in the valid sample	Greater than the 60th percentile of the length of stay of all patients in the valid sample	10

The overall severity score (OSS) was calculated based on the six severity variables using an empirical weight-driven additive model approach, as in Table 1. The OSS was converted into a categorical variable with patients less than or equal to 60 as not severe COVID-19 (NSC-19) and greater than 60 as severe COVID-19 (SC-19).

Statistical analysis plan

Exploratory Data Analysis

The data was explored using Python for initial understanding and overview. Relevant data cleaning and formatting were done. Records were double-checked for the inclusion and exclusion criteria. Records with missing data were excluded. Univariate and bivariate descriptive statistics were generated for all relevant fields (mean, standard deviation, interquartile ranges). Descriptive visualizations were made to understand the cross-correlations and patterns in the dataset.

Statistical Analysis

Logistic regression was used for creating six models: prediction of overall severity, prediction of death, prediction of shifting to the ICU, prediction of length of stay, prediction of oxygen requirement, and prediction of ventilator support. Modeling was not performed for the sixth severity measure (initial (direct) admission to ICU) as no additional prior data would be available for its practical applicability.

Ethical Consideration

The institutional review board reviewed, validated, and approved the study protocol (ethics approval number MH/2021/IEC).

## Results

**Figure 1:**
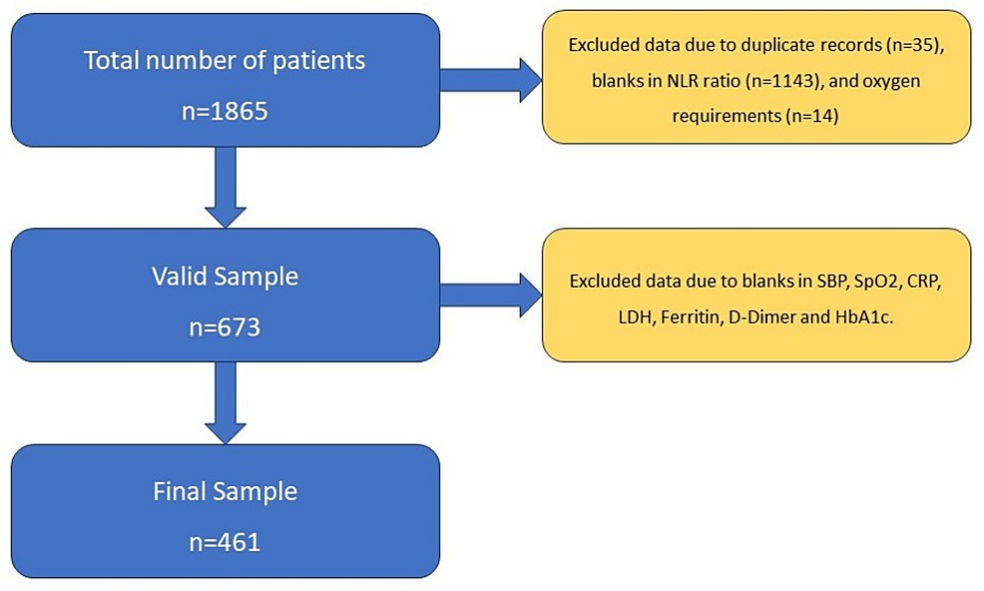
Flow chart of the sample NLR: neutrophil-lymphocyte ratio, SBP: systolic blood pressure, SpO2: oxygen saturation, CRP: C-reactive protein, LDH: lactate dehydrogenase, HbA1c: glycosylated hemoglobin

A total of 1,865 patient records were retrieved from the database. Only the records with at least three NLR values (baseline, 24 hours, and 48 hours) were included in the analysis sample. Out of the 1,865 patients, 35 patients were excluded with duplicate data, 1,143 patients had missing NLR ratio values of either day 1, day 2, or day 3, and 14 patients had missing details of oxygen requirement of either day 1, day 2, or day 3. From the remaining 673 patients, additional 212 records were removed because of missing SBP, SpO2, CRP, lactate dehydrogenase (LDH), serum ferritin, D-dimer, and HbA1c values. The final sample available for analysis was 461 patients (Figure 1).

The demographic and other details of the included patients are provided in Table 2.

**Table 2:** Demographics, SpO2, HbA1c, and comorbidities SC-19: severe COVID-19, NSC-19: not severe COVID-19, SpO2: oxygen saturation, HbA1c: glycosylated hemoglobin, CKD: chronic kidney disease, COPD: chronic obstructive pulmonary disease, CAD: coronary artery disease, CLD: chronic liver disease, CVA: cerebrovascular accident

	Overall (n = 461)	SC-19 (n = 64)	NSC-19 (n = 397)
Age group (years) (n(%))			
<=30 yrs	33(100)	2(6.06)	31(93.94)
31-40 yrs	89(100)	11(12.36)	78(87.64)
41-50 yrs	111(100)	13(11.71)	98(88.29)
51-60 yrs	95(100)	14(14.74)	81(85.26)
>60 yrs	133(100)	24(18.05)	109(81.95)
Gender (n(%))			
Male	316(100)	47(14.87)	269(85.13)
Female	145(100)	17(11.72)	128(88.28)
SpO2 (n(%))			
<90	38(100)	20(52.63)	18(47.37)
90-94	70(100)	16(22.86)	54(77.14)
95 and above	353(100)	28(7.93)	325(92.07)
HbA1c (mean(SD))	7.2(1.8)	7.3(1.8)	7.2(1.8)
Comorbidities (n(%))			
Diabetes mellitus	188(100)	21(11.2)	167(88.8)
Hypertension	174(100)	28(16.1)	146(83.9)
CKD	23(100)	5(21.8)	18(78.2)
COPD	4(100)	0	4(100)
Asthma	12(100)	0	12(100)
CAD	34(100)	10(29.4)	24(70.6)
Hypothyroidism	48(100)	8(16.7)	40(83.3)
CLD	1(100)	0	1(100)
CVA	9(100)	0	9(100)

About 397 patients were categorized as NSC-19 and 64 patients were categorized as SC-19, as discussed in the Materials and Methods section. The average age, SBP, DBP, SpO2, HbA1c, NLR, CRP, D-dimer, serum LDH, and serum ferritin of patients in the SC-19 and NSC-19 groups are given in Table 3. The ∆NLR (0-24 hours and 0-48 hours) was more in high-severity cases and was almost similar in low-severity cases (Table 3).

**Table 3:** Severity and inflammatory parameters ∆NLR: Delta neutrophil-lymphocyte ratio or change in neutrophil-lymphocyte ratio, SC-19: severe COVID-19, NSC-19: not severe COVID-19, BP: blood pressure, SpO2: oxygen saturation, HbA1c: glycosylated hemoglobin, NLR: neutrophil-lymphocyte ratio, CRP: C-reactive protein, LDH: lactate dehydrogenase

	Overall (n=461)	SC-19 (n = 64)	NSC-19 (n = 397)	p-value
	count (%)	count (%)	count (%)	
Age	51.3 (14.8)	55.2 (14.4)	50.6 (14.8)	0.022
BP - systolic	125.2 (51.7)	131.5 (16.6)	124.1 (55.3)	0.035
BP - diastolic	77.8 (7.2)	79.5 (10.0)	77.5 (6.6)	0.123
SpO2	95.1 (5.4)	89.8 (10.4)	95.9 (3.3)	<0.001
HbA1c	7.2 (1.8)	7.3 (1.8)	7.2 (1.8)	0.581
NLR Day1	5.7 (4.2)	8.4 (5.0)	5.3 (3.9)	<0.001
NLR Day2	6.1 (4.2)	9.5 (5.2)	5.6 (3.7)	<0.001
NLR Day3	6.3 (4.9)	11.1 (6.4)	5.5 (4.1)	<0.001
∆NLR (0-24 hours)	0.4 (3.5)	1.1 (4.9)	0.3 (3.2)	0.174
∆NLR (0-48 hours)	0.6 (4.2)	2.7 (5.7)	0.2 (3.8)	0.001
CRP (mg/L)	68.4 (71.4)	96.4 (83.0)	63.9 (68.4)	0.004
Serum LDH (U/L)	338.9 (101.8)	414.9 (57.3)	326.7 (102.1)	<0.001
Serum ferritin	242.4 (89.8)	286.1 (37.2)	235.3 (93.8)	<0.001
D-dimer levels (ng/mL)	350.1 (147.3)	432.9 (123.2)	336.7 (146.6)	<0.001

Stepwise logistic regression models were developed separately for the SC-19, death, oxygen requirement, ventilator support, ICU shifting, and length of stay. Here is a description of the most important variables for accurately predicting the overall disease severity, outcome (death), and resources (shifting to ICU, oxygen requirement, ventilator support, length of stay).

Overall severity prediction

**Figure 2:**
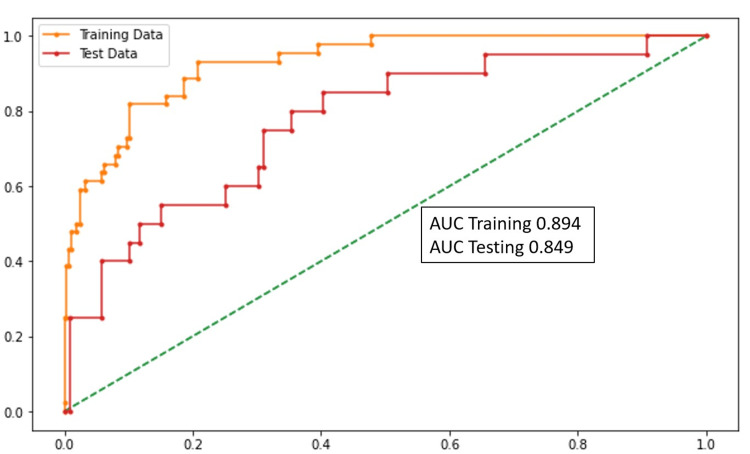
AUC graph (overall severity prediction) AUC: area under the curve

The variables that were statistically significant in the model for identifying SC-19 (patients who had an OSS of greater than 60) were SBP, SpO2, diabetes mellitus, patient history of coronary artery disease (CAD), ∆NLR (0-48 hours), LDH, and D-dimer levels. With the increase in ∆NLR, LDH, and D-dimer levels, the severity of the disease increases according to the prediction model. The performance of this model was assessed using the area under the curve (AUC), accuracy, sensitivity (which is a recall of true positives), and specificity (which is a recall of true negatives) and is highlighted in Figure 2 (accuracy, sensitivity, and specificity).

Death prediction

**Figure 3:**
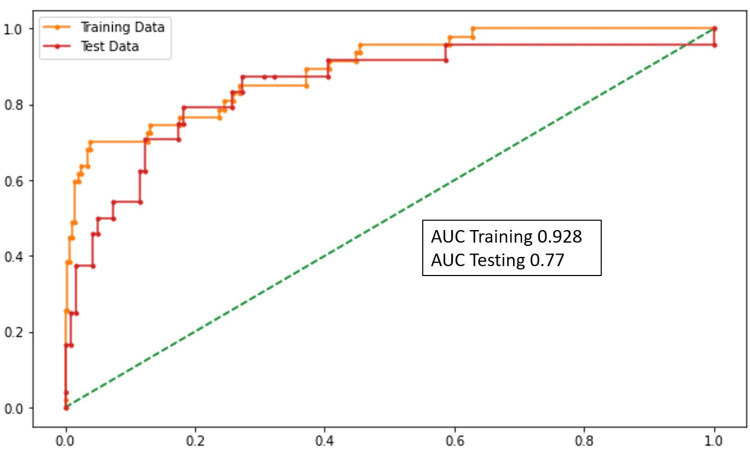
AUC graph (death prediction) AUC: area under the curve

SBP, SpO2, ∆NLR (0-48 hours), and LDH were the variables that were statistically significant in the model. According to the model, the increase in the blood pressure, ∆NLR (0-48 hours), LDH, and decrease in SpO2 predicts death in the patients. The performance of this model was assessed using the AUC (Figure 3).

Resources

Ventilator Support

**Figure 4:**
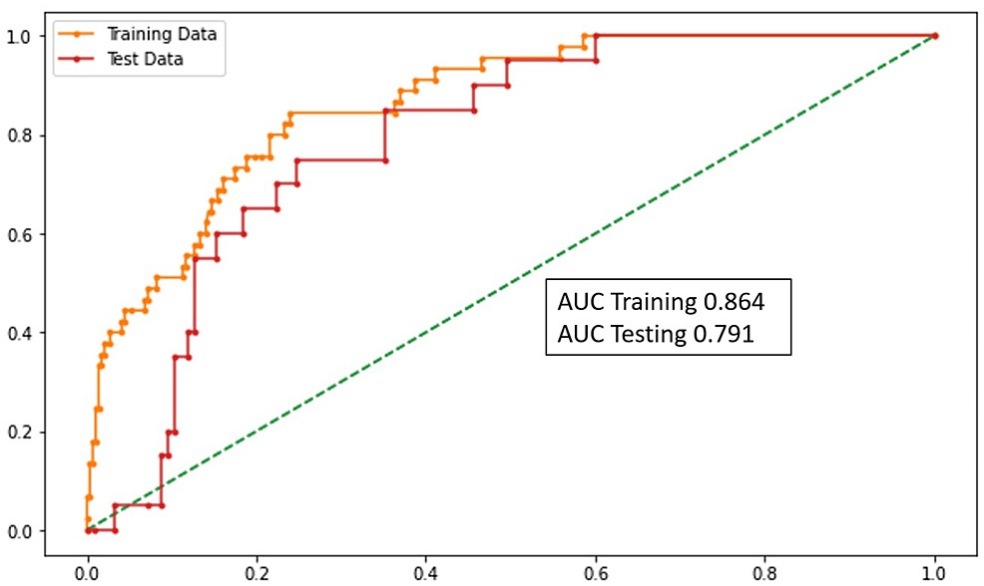
AUC graph (ventilator support) AUC: area under the curve

The age of the patient, chronic obstructive pulmonary disorder, hypothyroidism, shortness of Breath (SOB), ∆NLR (0-48 hours), and D-dimer levels were the statistically significant variables. According to the model, the need for ventilator support increased with the increase in ∆NLR and D-dimer levels. The performance of this model was assessed using the AUC (Figure 4).

Oxygen

**Figure 5:**
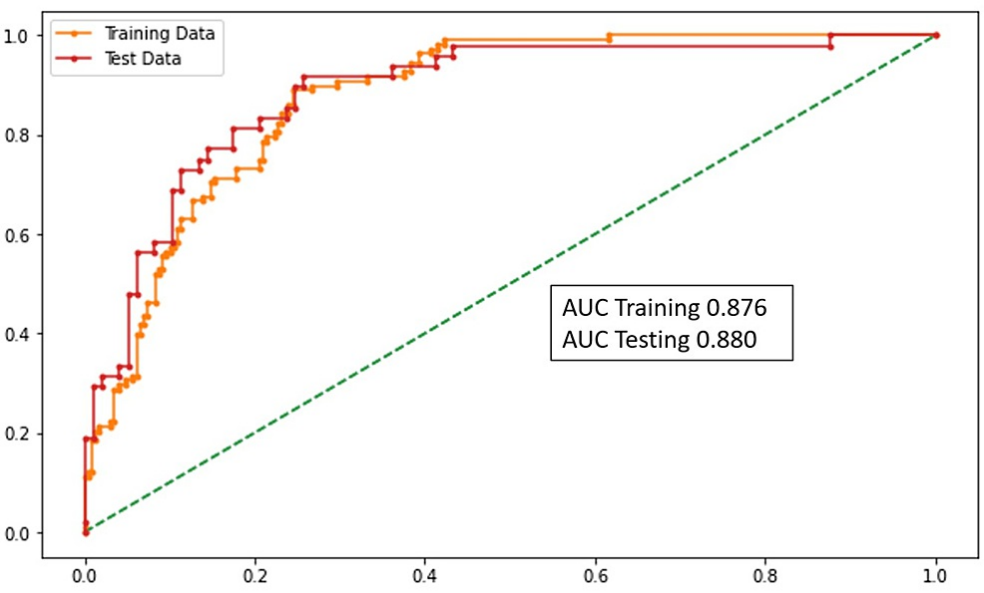
AUC graph (oxygen support) AUC: area under the curve

SpO2, SOB, oxygen severity, initial admission to ICU, ∆NLR (0-48 hours), LDH, and D-dimer levels were statistically significant. Based on this model, the patient was more likely to require oxygen support with reduced SpO2 and increased ∆NLR, D-dimer, and LDH values. The performance of this model was assessed using the AUC (Figure 5).

ICU Shifting

**Figure 6:**
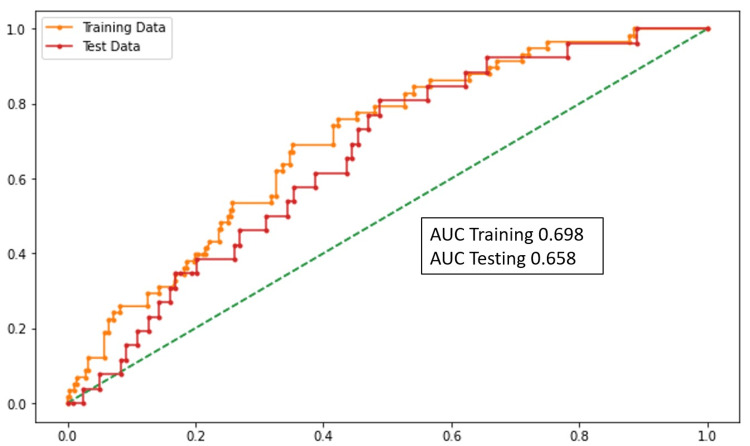
AUC graph (ICU shifting) AUC: area under the curve

∆NLR (0-48 hours) and D-dimer levels were significant. According to the model, with the increase in the ∆NLR and D-dimer, patients are more likely to be shifted to the ICU. The performance of this model was assessed using the AUC (Figure 6).

Length of Stay

**Figure 7:**
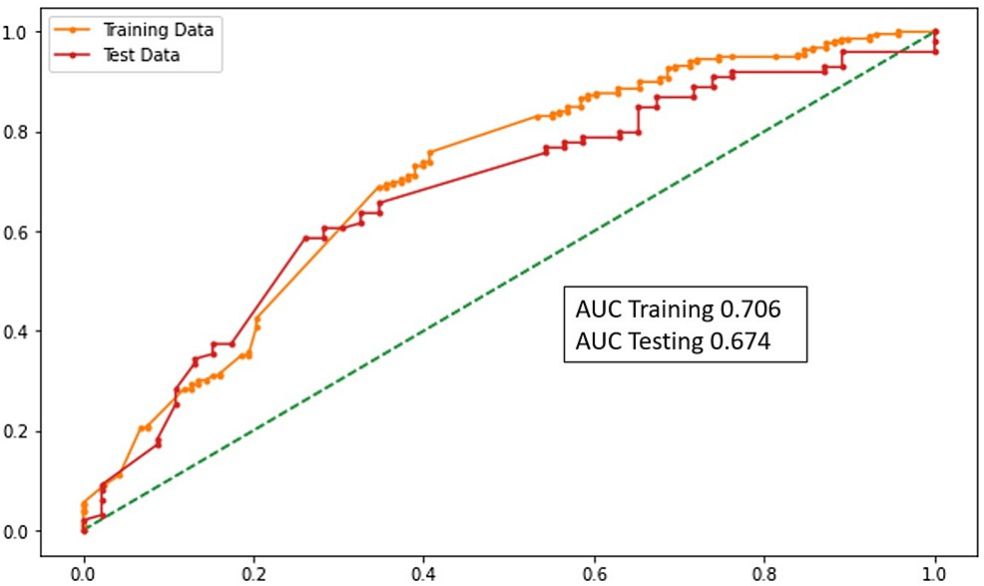
AUC graph (length of stay) AUC: area under the curve

Length of stay, oxygen requirement change (days 1-3), and serum ferritin were statistically significant. The performance of this model was assessed using the AUC (Figure 7).

**Figure 8:**
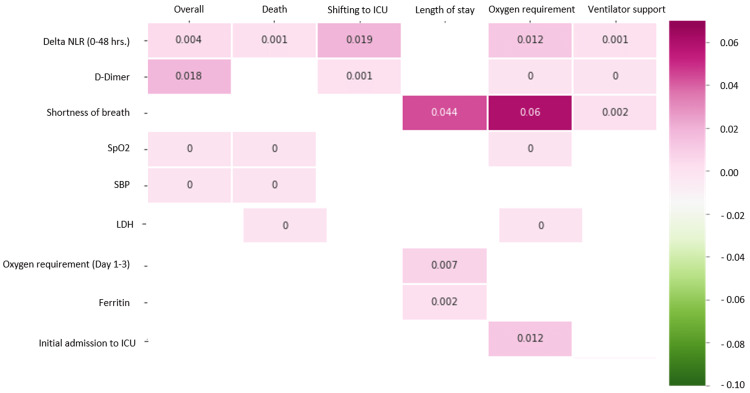
p-values of different variables ICU: intensive care unit, NLR: neutrophil-lymphocyte ratio, SpO2: oxygen saturation, SBP: systolic blood pressure, LDH: lactate dehydrogenase

The p-values and distribution of the different variables are highlighted in Figure 8.

**Figure 9:**
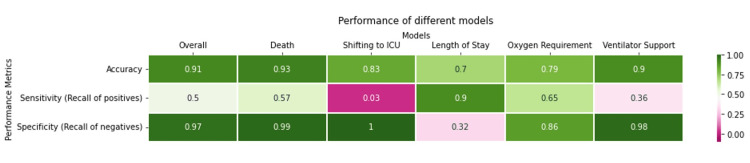
Performance of models ICU: intensive care unit

The performance of different models is given in Figure 9.

## Discussion

SARS-CoV-2 binds to alveolar angiotensin-converting enzyme 2 receptors, causing the production of inflammatory factors, which stimulate the immune system, resulting in a cytokine storm [31,32]. Hence, fast and precise identification of severe COVID-19 cases following diagnosis is critical. Many inflammatory indicators have been studied as predictors of mortality in COVID-19 hospitalized patients.

Hydrolysis of fibrin produces a degradation product called D-dimer [33]. It was reported by Ye et al. [34] that peak D-dimer value was significantly correlated with hospitalization days, and if the initial values are high, then the patient is at high risk of death and requires further intensive and immediate treatments. This study also showed that the AUC of D-dimer, peak D-dimer, was more than 0.7; thus, it is a good predictor of severity. A systematic review and meta-analysis conducted by Fialek et al. [35] in 264 records and Martha et al. [36] in 21 studies suggested that higher LDH values helped evaluate the severity and are associated with poor outcomes in COVID-19 patients. Increased CRP, D-dimer, ferritin, NLR, and LDH levels in the blood were linked to a greater risk of severe illness. The more severe the inflammatory condition, the higher the levels of inflammatory markers, and these indicators may assist in predicting severity and prognosis [37].

NLR is a well-documented biomarker of COVID-19 [38]. The ROC curve analysis in a study conducted by Sayah et al. [39] showed that NLR has the highest accuracy among hematological markers in assessing severity and mortality in COVID-19, with cut-off values of 5.9 and 7.4, respectively.

NLR has been studied extensively in the literature. A study by Ma et al. [40] suggested that NLR can distinguish between severe and non-severe cases of COVID-19. A meta-analysis conducted by Wang et al. [41] advised that the sensitivity, specificity, and AUC values of the NLR were 0.82, 0.77, and 0.87, respectively, indicating that it could distinguish severe cases from mild with high accuracy. Fifty-eight studies and 20 studies were included in a meta-analysis by Sarkar et al. [25] and Chan et al. [42], respectively, which revealed that severely ill patients are associated with increased NLR. A meta-analysis and various other studies [43,44] have predicted that an increased NLR on admission is associated with an increased risk for mortality. Maddani et al. [37] reviewed that high ferritin and NLR were independent predictors of the requirement of admission to the ICU. Tufa et al. [45], in their study, suggested that NLR is a biomarker with only modest accuracy for predicting disease severity and mortality.

∆NLR was defined as the difference between baseline and post-treatment NLR [28]. This study aimed to study the role of ∆NLR (0- 48 hours) in predicting the severity of COVID-19. We took various aspects of severity across six major domains (overall severity, death, ventilator support, oxygen, ICU shifting, and length of stay) and calculated single severity scores based on weighted scores, as explained earlier. As per our analysis, ∆NLR is statistically significant in predicting all five individual models and the OSS. Thus, ∆NLR is a valid marker for predicting severe cases of COVID-19. A study by Moisa et al. [46] stated that ∆NLR values >2 at 48 hours were the best independent predictor for invasive mechanical ventilation. It also stated that immunotherapy with tocilizumab appeared to reduce the ∆NLR values. According to a study conducted by Abensur Vuillaume et al. [47], the only biochemical factor significantly associated with mortality was again ∆NLR. It also stated that a positive ∆NLR (0-24 hours) was associated with poor prognosis. The clinical relevance of ∆NLR is a more comprehensive discriminator and is significant across all four domains and the overall severity. Compared to other parameters, ∆NLR is the only significant parameter in all the abovementioned prediction models. We recommend that ∆NLR can be used to evaluate the severity of COVID-19 and many other disorders. It can be used as a promising predictor of severity in other acute inflammatory and cytokine-mediated disorders.

The present study is a first of its kind in India, and the strengths of this study include adequate sample size, good data collection, use of logistic regression and individual models, and use of different biomarkers in the same patients. Patients with COVID-19 were diagnosed and provided treatment according to the standard of care. An observational study was conducted with a sample size of 1,865 patients, and patient selection criteria were clearly demonstrated. The data were cleaned, multiple biomarkers similar to ∆NLR were identified, and their predictive capabilities were compared.

This study also presented a direct comparison of the predictive relevance of biomarkers (NLR, ∆NLR, D-dimer, CRP, LDH, ferritin) that represent the body’s inflammatory response and sound statistical concepts that included a logistic regression to create independent models of severity. Furthermore, this study also presented an extensive review of literature documenting the raising concerns of the limited applicability of baseline NLR and papers documenting the role of the advantages of the ∆NLR.

The main limitation of the study was that cytokines like interleukin-2 receptor (IL-2R) and IL-6 were not tested as they are not cost-effective, and the benefits of cytokines are still controversial.

## Conclusions

This study presents a unique biomarker. ∆NLR, according to this study and analysis, is a significant predictor of the severity of COVID-19. Although NLR at baseline is an extensively studied biomarker, its redundancy as a sole marker is questionable. Its limitation is also palpable with recent publications demonstrating only a modest applicability of NLR alone in predicting disease severity. This study applied the concept of ∆NLR that has been reported in only four or five publications in patients with COVID-19.

Estimating the severity of COVID-19 is an essential parameter at both individual patient and population levels. It can help the clinician in timely escalating the treatment strategy, titrating the management with increasing cytokine storm, and also help the epidemiologist or hospital managers to plan and manage resources. ∆NLR and other inflammatory parameters predict the prognosis, severity, and mortality due to the infection. However, its scope of use in other disorders’ prognoses must be further researched. Including these in decision-making strategies result in more effective and timely management of inflammation-mediated fatal disorders.

## Figures and Tables

**Figure 10:**
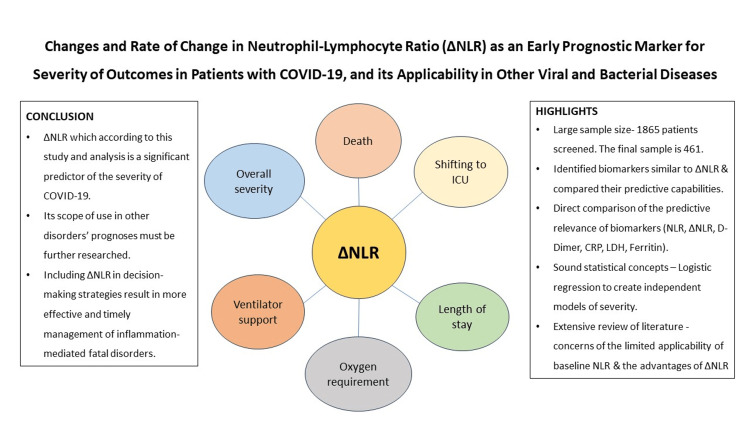
Visual abstract ∆NLR: Delta neutrophil-lymphocyte ratio or change in neutrophil-lymphocyte ratio, ICU: intensive care unit, CRP: C-reactive protein, LDH: lactate dehydrogenase

**Video 1:** Article highlights by Dr. Nandana

**Figure 11:**
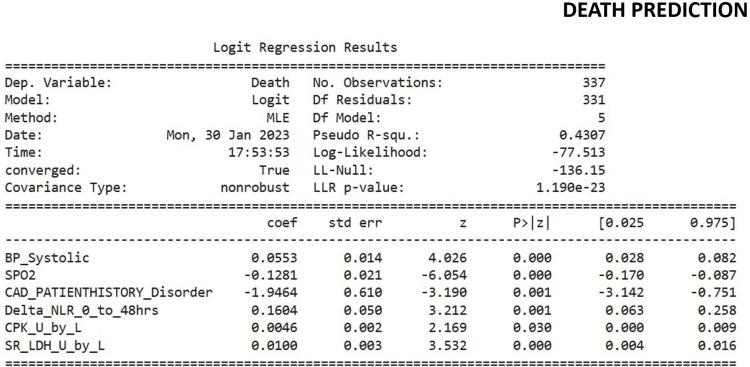
Death prediction (logistic regression model)

**Figure 12:**
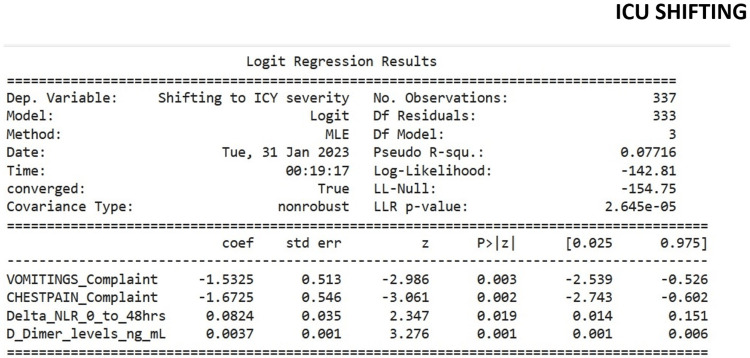
ICU shifting (logistic regression model)

**Figure 13:**
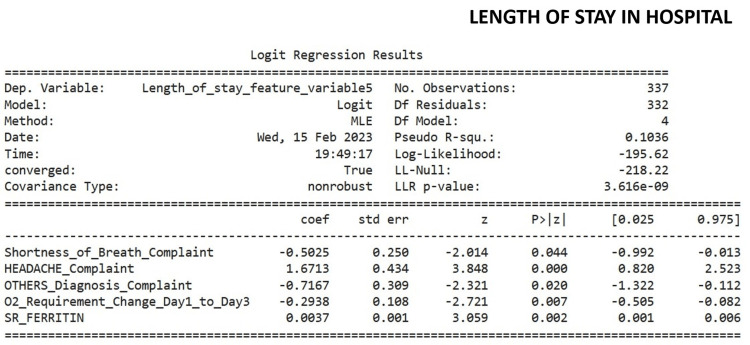
Length of stay (logistic regression model)

**Figure 14:**
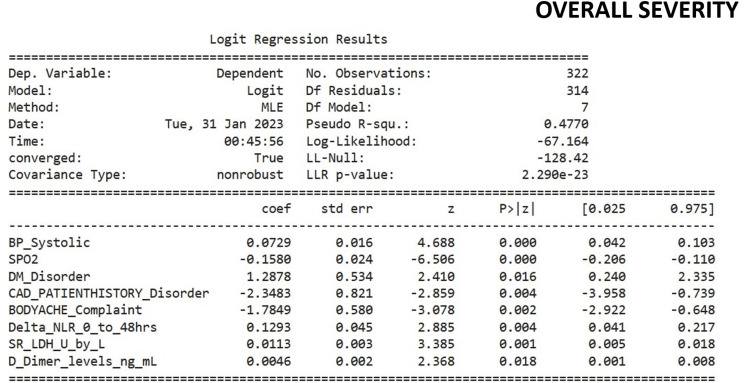
Overall severity (logistic regression model)

**Figure 15:**
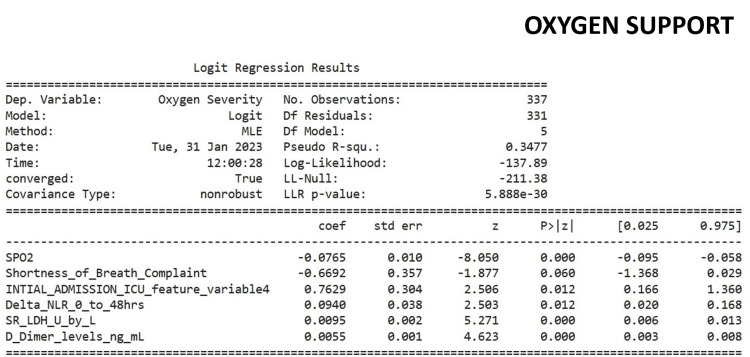
Oxygen support (logistic regression model)

**Figure 16:**
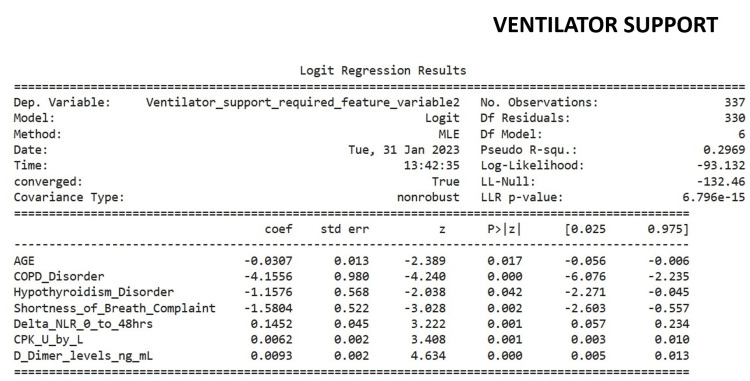
Ventilator support (logistic regression model)
